# Diabetes Control and the Occurrence of Postoperative Hyperglycemia Among Adults With Type 1 and Type 2 Diabetes in a Tertiary Care Center

**DOI:** 10.1155/ije/8829049

**Published:** 2025-09-15

**Authors:** Hanan Aljedani, Jomanah Mazi, Arwa Almutairi, Halah Namnkani, Reffal Aldainiy, Suhaib Radi, Alaa Althubaiti

**Affiliations:** ^1^College of Medicine, King Saud Bin Abdulaziz University for Health Sciences, Jeddah, Saudi Arabia; ^2^Department of Research, King Abdullah International Medical Research Center, Jeddah, Saudi Arabia; ^3^Department of Internal Medicine, Division of Endocrinology, Ministry of the National Guard-Health Affairs, Jeddah, Saudi Arabia

**Keywords:** diabetes mellitus, intra-abdominal surgery, orthopedic surgery, postoperative infections, postoperative hyperglycemia

## Abstract

**Background:** Diabetes mellitus (DM) is a growing concern globally. DM control is indicated by hemoglobin A1c, measuring glucose levels within two to 3 months. Patients with DM who have surgery may experience postoperative hyperglycemia (POHG) which is associated with many complications. This study aimed to investigate POHG incidence among patients with DM based on their level of glycemic control.

**Methods:** This was a retrospective cohort study of patients with DM ≥ 18 years who had orthopedic, intra-abdominal, cardiothoracic, or vascular surgery at King Abdulaziz Medical City, Jeddah, between October 2016 and October 2023. Patients with DM were considered controlled if the hemoglobin A1c was < 7%.

**Results:** The study included 306 patients, and the majority (69.28%) experienced POHG. There was a significant association between POHG and the level of glycemic control. POHG was experienced by 32.55% of patients with controlled DM vs 67.45% of patients with uncontrolled DM. Furthermore, patients with preoperative random glucose readings (RBG) of ≥ 9.2 mmol/L had a significantly higher risk of developing POHG. Moreover, older age and male sex were associated with higher POHG risk.

**Conclusion:** Our data indicate that the incidence of POHG was significantly greater among patients with uncontrolled DM. Patients with DM with a preoperative RBG of ≥ 9.2 mmol/L had a higher likelihood of developing POHG. Future research should include a larger sample and investigate associations between POHG, other complications, and the influence of varying levels of DM control.

## 1. Introduction

Diabetes mellitus (DM) is one of the most common metabolic conditions, either a lack of insulin production or the body's inability to respond to insulin [1]. This causes a rise in blood glucose levels, which can affect numerous organ systems leading to neuropathy, peripheral artery disease, and end-stage renal disease, particularly if blood glucose levels are poorly controlled [2]. Furthermore, DM is a known risk factor for postoperative hyperglycemia (POHG) [3]. Hospitals typically order hemoglobin A1c (HbA1c) tests to evaluate average blood glucose levels over the previous two to 3 months, which indicates DM level of control [4]. Those with an HbA1c level of < 7%, well-controlled DM, are less likely to have a poor surgical outcome [2, 5].

According to the World Health Organization (WHO), the number of people with DM worldwide increased from 422 to 424.9 million between 2014 and 2017, representing a global prevalence of 8.8%. As a result, the International Diabetes Federation has estimated that the number of adults with DM will reach 693 million in 2045, which is 9.9% of the world's population [6]. The incidence of POHG in patients admitted to surgical units ranges from 16% in people without DM to 24% in those with DM, with incidence rates dependent on the type of surgery performed, patient comorbidities, hospital facilities and catchment area, and the nature of the disease being treated [7]. Hospitalized patients with hyperglycemia have an increased risk of complications and mortality, higher rates of ICU admissions, and an increased risk of postoperative surgical site infection (SSI) [8]. The relationship between POHG and SSI has been reported in several studies, including orthopedic and lower extremity procedures [3, 9].

DM is widespread in the Kingdom of Saudi Arabia (KSA) [10], and the WHO has ranked its prevalence in KSA as the second highest in the Middle East and the seventh worldwide [11]. Also, it has been reported that more than half of the Saudi population aged ≥ 30 years have blood glucose levels that meet either DM or pre-DM criteria [12]. Unfortunately, DM was responsible for > 10% of deaths in Arab region [13]. In addition, unfavorable postoperative outcomes are common among patients with DM in KSA, with 21.5% developing complications, such as SSIs [14]. While some studies have identified DM as an independent risk factor for postoperative infection [15, 16], a study examining infections following cardiac surgery reported that DM was not significantly associated with sternal wound infection [17].

Patients with DM who require surgery can experience persistently hyperglycemia, which may necessitate careful monitoring and control throughout the perioperative period [18]. This study aimed to investigate the incidence of POHG in patients with DM and compare its rate based on DM control with the hypothesis of increased risk of POHG in uncontrolled DM patients.

## 2. Materials and Methods

### 2.1. Subjects and Study Settings

This was a retrospective cohort study of patients with DM who had orthopedic, intra-abdominal, cardiothoracic, or vascular surgery at the National Guard Health Affairs (NGHA) at King Abdulaziz Medical City in Jeddah between October 2016 and October 2023. Medical students collected the data under the supervision of the research supervisor. A total of 306 medical records were collected from the BestCare system, BestCare 2.0, developed by Seoul National University Bundang Hospital, which is used in the NGHA facility to store electronic medical records.

The inclusion criteria for the study were as follows: patients with Type 1 or Type 2 DM ≥ 18 years of age underwent blood glucose testing during admission and after surgery and had HbA1c results available before surgery or within the first 7 days of admission. Individuals were excluded if they had active preoperative infections, incomplete medical records, preoperative coma, needed an emergent procedure, required mechanical ventilation, preoperative sepsis, receiving corticosteroids, previous amputation, wounds with obvious signs of preoperative infection, active foot ulcers with exposed bone and underwent reconstruction, gestational diabetes, or multiple surgeries. Consecutive sampling was applied to select the study population. A complete list of all patients who underwent the specified types of surgeries during the study period was obtained. Records were then reviewed consecutively, starting from the beginning of the list, until the targeted sample size was reached. This method ensured that every eligible patient had an equal chance of being included, minimizing selection bias.

The primary objectives were to: assess the association between the level of diabetes control and the incidence of POHG among patients with DM, compare rates of POHG between patients with well-controlled and poorly controlled DM, identify any association between type of DM and POHG, and evaluate POHG incidence following each type of surgery. The study also aimed to investigate four secondary objectives: (1) assess the rate of postoperative infections in patients with DM; (2) identify which DM type is associated with risk of developing postoperative infection; (3) compare postoperative infection rates between individuals with well-controlled and poorly controlled DM; and (4) investigate the relationship between POHG and postoperative infection.

### 2.2. Data Collection

Data were collected following chart review, with variables divided into patient information, demographic data, and surgical information. Patient information and demographic data included: serial number (a number given to each patient to ensure confidentiality), age, sex, smoking status, weight (kg), BMI (kg/m^2^), HbA1c levels (preoperatively or within 7 days of admission), DM type, DM duration (years), and medications for DM used at home, including insulin, metformin, sulfonylurea, dipeptidyl peptidase 4 inhibitors, glucagon-like peptide 1 agonists, and sodium-glucose co-transporter 2 inhibitors. The following comorbidities were recorded: hypertension, dyslipidemia, cardiac disease, cancer, stroke, diabetic neuropathy, diabetic nephropathy, diabetic retinopathy, peripheral vascular disease, obesity, arthritis, and end-stage renal disease.

Surgical information included type of surgery, length of hospital stay postoperatively (days), place of postoperative admission (critical care, ward), POHG occurrence, average preoperative random blood glucose (RBG) and fasting blood glucose (FBG) during admission, number of preoperative days with average readings > 10 mmol/L, average postoperative RBG and FBG during admission, number of postoperative days with average readings > 10 mmol/L, inpatient treatment, occurrence of postoperative SSI, time until development of postoperative infection (days), focus of infection (skin, deep abscess, lungs, urinary tract, joints, central nervous system, gastrointestinal tract), time until resolution of infection (days), and infection-related death.

Patients were classified as having controlled DM if their HbA1c was < 7%. In case HbA1c was not available, the level of control was determined using preoperative glucose measures. Since the study was focused on preoperative and postoperative glucose measures and type of surgery, records were considered incomplete if any of these variables were missing. Patients were considered to have POHG if there were one or more postoperative days with glucose levels > 10 mmol/L.

### 2.3. Data Analysis

JMP, Version 17 (SAS Institute Inc., Cary, NC, 1989–2023) was used for the analysis. The Shapiro–Wilk test was used to assess normality. Chi-square and Wilcoxon tests were used to analyze nonparametric data, and *t*-tests were used for parametric data. Descriptive data included numbers and percentages. The median and interquartile range (IQR) were calculated for nonparametric data, while the mean and standard deviation (SD) were calculated for parametric data. Multiple logistic regression analysis was performed to determine the relationship between POHG and other significant variables. Odds ratios and 95% confidence intervals were calculated. The level of significance was set at *p* < 0.05.

### 2.4. Ethical Considerations

No consent form was needed since data were collected retrospectively using chart review. The study was approved by King Abdullah International Medical Research Center (IRB/2821/22).

## 3. Results

### 3.1. Patient Demographics

The median age of the population was 63.5 years (IQR = 18). More than half were women (*n* = 177, 57.84%), and 42.15% were men (*n* = 129). Most of the study population had Type 2 DM (*n* = 273, 89.21%). About half of the patients (*n* = 135, 44.11%) had well-controlled DM and 171 (55.88%) had poorly controlled DM. Hypertension was the most common comorbidity, with more than two-thirds of participants affected (*n* = 216, 70.59%) followed by dyslipidemia (*n* = 105, 34.31%), cardiac disease (*n* = 44, 14.38%), and cancer (*n* = 32, 10.46%). Insulin was the most frequently used medicine for DM by patients at home and during hospitalization, utilized by 103 (33.66%) and 179 (58.49%) patients, respectively.

Among the 306 patients, 126 (41.17%) had intra-abdominal surgery, 122 (39.86%) had orthopedic surgery, 45 (14.7%) had cardiothoracic surgery, and 13 (4.25%) had vascular surgery. Most patients had open surgery (*n* = 263, 85.94%), while 43 (14.05%) had laparoscopic procedures. POHG was present in 212 (69.28%) of the study's participants. Following surgery, the median (IQR) length of hospital stay was 3 (3) days. Moreover, 276 (90.19%) patients went to the ward, while only 30 (9.8%) went to the critical care unit. The median (IQR) preoperative RBG level was 9.34 (4.55) mmol/L, while the postoperative value was 9.97 (4.49) mmol/L. The median (IQR) preoperative FBG level was 8.7 (6.1) mmol/L, and the postoperative value was 9.5 (5.49) mmol/L.

Postoperative infection was present in only a small number of patients (*n* = 9, 2.94%). Postoperative infection required a mean (SD) of 9 (7.14) days to develop postoperatively, and all infections were treated and subsequently resolved in the hospital. Among patients with infections, 7 (77.8%) developed SSI, while 2 (22.2%) had an infection unrelated to the surgical site. Specifically, 3 (33.3%) patients had a skin infection, 3 (33.3%) had a deep abscess, 2 (22.2%) had a urinary tract infection, and one (11.1%) had a gastrointestinal infection. The median time until infection resolution was 17 days (IQR 54.25). Three patients were readmitted with postoperative infection, yet there were no infection-related deaths.

### 3.2. POHG and Other Factors

Age, sex, and BMI were significantly associated with POHG (Table 1). In the group of patients with POHG, 47.64% were males compared to 29.79% in the group without POHG (*p*=0.003). In addition, the median age was higher in patients with POHG than in those without POHG (65 vs. 57.5 years, *p*=0.01). In contrast, median BMI was significantly lower in the group with POHG compared with the group without POHG (29.83 vs. 32.88, *p* < 0.001). However, none of these factors were found to significantly affect the presence of POHG.

There was a statistically significant difference between the number of patients with POHG who had well-controlled vs uncontrolled DM (32.55% vs. 67.45%, respectively, *p* < 0.001). As shown in Table 1, POHG was more common in the group with poorly controlled diabetes. There was no significant association between diabetes type and POHG (*p*=0.46). POHG was most common in patients who underwent orthopedic surgery (45.75%) followed by intra-abdominal (33.96%), cardiothoracic (16.04%), and vascular surgeries (4.25%). However, POHG was significantly associated only with orthopedic and intra-abdominal surgeries (*p*=0.002 and *p* < 0.001, respectively). The median length of hospital stay was significantly longer in patients with POHG than in those without (4 [IQR 4] days vs. 3 [IQR 2] days, *p*=0.004) (Table 2).

### 3.3. Blood Glucose Measurements

There was a statistically significant association between HbA1c and POHG (*p* < 0.001). Patients with POHG had a higher median (IQR) HbA1c % than those without POHG (7.9 [2.55] vs. 6.4 [1.23]), respectively. The median (IQR) preoperative RBG and FBG in patients with POHG were 10.52 (5.08) mmol/L and 9.53 (6.33) mmol/L, respectively, compared with 7.71 (2.49) mmol/L and 6.8 (3.6) mmol/L, respectively, in patients without POHG (*p* < 0.001 for both comparisons). The median (IQR) postoperative RBG and FBG in patients with POHG were 11.3 (3.72) mmol/L and 10.7 (5.2) mmol/L, respectively, compared with 7.25 (1.87) mmol/L and 7.1 (2.6) mmol/L in patients without POHG (*p* < 0.001 for both comparisons), respectively. Patients with POHG spent a median (IQR) of 1 (2) preoperative days and 2 (3) postoperative days with an average blood glucose level of > 10 mmol/L. In contrast, patients without POHG spent a median (IQR) of 0 (0.25) days preoperatively (*p* < 0.001) (Table 2).

### 3.4. Infection and Other Factors

Most patients with postoperative infections had Type 2 DM, accounting for 88.89% of the total study population (Table 3). Examining the relationship between postoperative infection and glycemic control, six (66.67%) patients with uncontrolled DM had a postoperative infection, compared with only three (33.3%) patients with controlled DM. Six of 212 (2.83%) patients with POHG developed postoperative infections compared with 3 of 94 (3.19%) patients without POHG. However, when the relationship between postoperative infection and DM type, level of control, and presence of POHG was examined, no statistically significant associations were found with *p* values of 0.97, 0.51, and 0.86, respectively (Tables 2 and 3).

**Figure 1:**
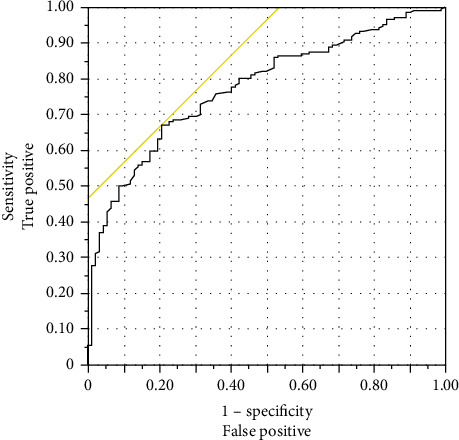
Receiver-operating characteristic curve of average preoperative random blood glucose during admission. The area under the curve value for the average preoperative random blood glucose level was 0.77. The cutoff value was 9.2 mmol/L.

A multiple logistic regression analysis was performed to investigate the relationship between POHG and other independent factors (Table 4). The results showed a statistically significant correlation between POHG and two factors: level of control (OR, 1.99; 95% CI, 1.05–3.75; *p*=0.035) and preoperative RBG (OR, 1.28; 95% CI, 1.11–1.50; *p*=0.001). Then, the predictive value of the average preoperative RBG level during admission for POHG was investigated. The area under the curve value for the average preoperative RBG level was 0.77, with a cutoff value of 9.2 mmol/L (Figure 1).

## 4. Discussion

POHG is frequently seen in individuals with and without DM, and it has been associated with a variety of complications, such as greater mortality risk, more intensive care unit admissions, and an increased risk of postoperative SSI [8, 18]. However, to the authors' knowledge, no prior research has analyzed POHG in patients with DM according to glycemic control, particularly in KSA. Consequently, our study aimed to investigate the impact of DM control on the occurrence of POHG. We found a statistically significant association between POHG incidence and DM control, with POHG being more common in patients with uncontrolled DM.

Conversely, patients with well-controlled DM had a lower incidence of POHG. Other findings in this study included associations between POHG and orthopedic and intra-abdominal surgery, male sex, older age, lower BMI, higher HbA1c levels, longer hospital stays, higher RBG and FBG levels, and a greater number of days with average glucose levels above 10 mmol/L. No significant associations were found between infection and level of control, DM type, or POHG.

One of our objectives was to determine the incidence of POHG in patients with DM. Of the 306 patients in the study, the majority (69.28%) had POHG. This is at the higher end of the rates reported in the literature (25% and 70%) [19, 20]. Even in patients without diabetes, the POHG rates were as high as 25%–55% in some studies [20, 21], whereas another study reported higher rates of POHG [22]. While we found a significant association between the incidence of POHG and the level of DM control, we found no similar results when searching the literature. However, one study reported an association between decreased postoperative complication and good glycemic control [23]. Furthermore, one study noted that the higher the preoperative blood glucose (normoglycemia vs. prediabetes vs. diabetes), the higher the rate of POHG [20]. The wide variation in the reported rates of POHG, ranging from 25% to 70% across studies, can largely be attributed to differences in study populations. Geographic variability likely reflects differences in cultural practices, dietary patterns, and health literacy, all of which influence glycemic control and postoperative outcomes. Moreover, the incidence of POHG has been shown to vary substantially, depending on institutional characteristics, patient comorbidities, and the type of surgical procedures performed. For example, literature reports POHG rates as low as 22% in vascular surgeries and as high as 77% in cardiac surgeries. These disparities highlight the importance of considering both methodological heterogeneity and population differences when comparing POHG rates across studies. Notably, evidence supports that effective perioperative and intensive care unit glycemic control, especially strategies that minimize glycemic variability, can lead to improved postoperative outcomes [7].

Our research showed that patients with RBG levels of ≥ 9.2 mmol/L are more likely to develop POHG. To the best of our knowledge, no research has determined the precise threshold for increased POHG risk. However, we investigated the relationship between POHG, as a postoperative complication, and specific preoperative glucose readings. We found a similar study in which patients were classified based on their preoperative hyperglycemia into mild (≥ 7.8 mmol/L), moderate (≥ 10 mmol/L), and severe (≥ 13.9 mmol/L). Postoperative complications linked with each group were then investigated. Univariate analysis in patients with and without DM revealed an association between postoperative complications and mild or moderate preoperative hyperglycemia. However, the multivariate analysis showed that only moderate preoperative hyperglycemia in patients with DM was significantly linked to postoperative complications [24]. Based on this classification, our study found a significant association with mild preoperative hyperglycemia. This might be because the authors of the previous study examined immediate glucose levels only within 6 h of surgery, whereas we included glucose levels from at least 3 days preoperatively.

We investigated the association between DM type and POHG since it had not previously been studied. However, there was no difference in the incidence of POHG between types of DM. This might be because of the imbalance in numbers between the two groups. Consequently, further research involves an equivalent number of individuals in each group.

Our results demonstrated that POHG was significantly associated with intra-abdominal and orthopedic surgeries. As far as we are aware, no prior research has investigated this topic. However, it has been reported that hyperglycemia following major clinical episodes, such as trauma or surgery, is a common response to hypermetabolic stress, which increases the hepatic glucose synthesis and decreases cellular glucose uptake to provide additional energy, but the sharp spike might have complications. In addition, inadequate glucose control prior to this response, such as in patients with DM, would contribute to the development of hyperglycemia [18]. Furthermore, we linked POHG to longer hospital stays, which is consistent with other studies [23, 25].

Our study could not establish a significant association between POHG and SSI. In contrast, one study found that patients with POHG had higher infection rates (23%) than those without POHG (14%), while DM alone was not associated with increased infection [3]. A further two studies reported the same association between POHG and SSI, but also reported an association between DM and infection [23, 26]. SSI in patients with DM varies from study to study. Unlike our study, comparing SSI in individuals with well-controlled and poorly controlled DM, many studies compared SSI in people with and without DM. One study reported a 33.3% incidence of SSI in people with DM; however, only nine individuals in the study population had DM [27]. Another study that included 39 patients with DM reported the SSI rate of 10.3% [28]. Nevertheless, we found an SSI rate of only 2.94%, which is clearly lower than both previous studies, despite including much higher numbers of individuals with DM (*n* = 306). However, our findings are consistent with the total incidence of SSI in KSA regardless of the glycemic status (0%–32.2%) [29]. Additionally, the low number of postoperative infections observed in our study may be attributed to several factors. Infection was defined by both positive culture results and the presence of clinical symptoms, which may have led to underreporting of milder or subclinical cases. Additionally, hospital policy emphasizes early discharge once patients are clinically stable to minimize the risk of hospital-acquired infections, which may have reduced the likelihood of detecting postoperative infections during hospitalization. However, this approach also increases reliance on outpatient follow-up, which may be inconsistent, particularly for patients from remote areas with limited healthcare access. Variations in health literacy may further affect patients' understanding of discharge instructions and their ability to recognize or report postoperative complications.

Our study also found no statistically significant difference in postoperative infections between people with well-controlled and poorly controlled diabetes, yet many studies reported an associations between POHG, HbA1c, and the risk of developing SSIs [9, 30–32]. However, one study stated that HbA1c ≥ 11.5% was not a predictor of SSI risk [33]. Additionally, although there was no significant association between SSI and DM type in our study, another study found that patients with Type 2 DM are more prone of developing SSI; however, the association became insignificant in the multivariate analysis [34].

Our findings could be explained by the inclusion of only patients with DM; in contrast, previous studies included patients with and without DM. A possible explanation for the impact of including those without DM is the diabetes paradox. This states that among individuals with hyperglycemia, those without DM are more likely to develop complications than those with DM. Possible explanations for poor outcomes in individuals without DM are as follows: (1) development of hyperglycemia in patients without DM may indicate greater surgical stress which causes previously undetected DM to become apparent during the perioperative period; (2) patients with DM who experience hyperglycemia perioperatively are more likely to receive insulin than those without DM; (3) insulin may be poorly tolerated in patients who have never took insulin previously, including those without DM and those with DM who are not using insulin at home; and (4) although acute hyperglycemia may have negative effects, persistent hyperglycemia may provide some protection against hypoxia-related diseases in individuals with DM since their bodies are accustomed to frequent high blood glucose levels [35]. Another explanation is that prior studies reporting a link between POHG and other complications studied populations of more than 1000 patients with and without DM [3, 12, 23, 26]. A greater number of patients could have provided these studies with a more diverse range of individuals with varying lengths of illness, environments, and responses to surgeries. Furthermore, the precautionary strategies employed at the NGHA hospital could have helped lower the incidence of POHG complications.

Our study focused on patients with DM and their level of glycemic control since the effect of control on POHG has not been well studied previously. We believe our findings might be the first step toward a larger scope of research investigating the relationship between glycemic control and additional postoperative complications. However, the postoperative infection rate was low in our study, making it difficult to find statistically significant differences.

The study had several limitations, including the relatively small sample size and limited availability of certain clinical data. These factors may have affected the ability to detect significant associations, particularly regarding POHG and postoperative infections. Specifically, the low number of postoperative infections (nine cases) limited the statistical power to detect a significant association between POHG and infection. Nonetheless, our findings support the study hypothesis, suggesting a decreased incidence of POHG among patients with effective diabetes control. However, to better evaluate the relationship between POHG and postoperative complications—particularly infections—future research should include larger, more diverse patient populations and settings. This would allow for more robust analyses and improve generalizability. Additionally, multicenter collaboration could enhance the sample size and variability in hospital practices, helping to identify more effective protocols for minimizing postoperative complications.

## 5. Conclusion

The incidence of POHG varies among patients with DM based on their level of glycemic control, and this study suggests that individuals with well-controlled DM have a lower risk of POHG. A greater risk of developing POHG was seen in patients with DM with a preoperative RBG level ≥ 9.2 mmol/L. Future research should include more participants and investigate potential associations between POHG and other complications, as well as the influence of varying levels of glycemic control.

## Figures and Tables

**Table 1:** Patient demographics, glycemic control, type of diabetes, and type of surgery according to POHG status.

	POHG (*n* = 212)	No POHG (*n* = 94)	*p* value
Age (years)			
Median (IQR)	65 (16.00)	57.5 (21.5)	0.010
Sex			
Male, *n* (%)	101 (47.64%)	28 (29.79%)	0.003
Female, *n* (%)	111 (52.36%)	66 (70.21%)	
BMI (kg/m^2^)			
Median (IQR)	29.83 (9.02)	32.88 (9.02)	< 0.001
Home medication (yes)^∗^			
Insulin, *n* (%)	77 (36.32%)	26 (27.66%)	0.14
Metformin, *n* (%)	44 (20.75%)	23 (24.47%)	0.47
Sulfonylurea, *n* (%)	13 (6.13%)	3 (3.19%)	0.29
GLP1 agonists, *n* (%)	4 (1.89%)	1 (1.06%)	0.60
DDP4 inhibitors, *n* (%)	10 (4.72%)	1 (1.06%)	0.11
SGLT2 inhibitors, *n* (%)	4 (1.89%)	0 (0.00%)	0.18
Comorbidities			
Hypertension, *n* (%)	151 (71.23%)	65 (69.15%)	0.71
Dyslipidemia, *n* (%)	74 (34.91%)	31 (32.98%)	0.74
Cardiac disease, *n* (%)	33 (15.57%)	11 (11.70%)	0.37
Cancer, *n* (%)	20 (9.43%)	12 (12.77%)	0.38
Level of control			
Controlled, *n* (%)	69 (32.55%)	66 (70.21%)	< 0.001
Uncontrolled, *n* (%)	143 (67.45%)	28 (29.79%)	
Type of diabetes			
Type 1	21 (9.91%)	12 (12.77%)	0.46
Type 2	191 (90.09%)	82 (87.23%)	
Type of surgery			
Orthopedic, *n* (%)	97 (45.75%)	25 (26.60%)	0.002
Intra-abdominal, *n* (%)	72 (33.96%)	54 (57.45%)	< 0.001
Cardiothoracic, *n* (%)	34 (16.04%)	11 (11.70%)	0.32
Vascular, *n* (%)	9 (4.25%)	4 (4.26%)	0.99

*Note:* DDP4 inhibitors = dipeptidyl peptidase 4 inhibitors, GLP1 agonists = glucagon-like peptide-1 agonists, SGLT2 inhibitors = sodium-glucose co-transporter-2 inhibitors.

Abbreviations: IQR = interquartile range, POHG = postoperative hyperglycemia.

^∗^Total patients who took home medication 161.

**Table 2:** Admission-related information, infection rates, blood glucose measurements, and inpatient treatment according POHG status.

	POHG (*n* = 212)	No POHG (*n* = 94)	*p* value
Place of admission			
Critical care, *n* (%)	24 (11.32%)	6 (6.38%)	0.18
Ward, *n* (%)	188 (88.68%)	88 (93.62%)	
Length of hospital stay (days)			
Median (IQR)	4 (4)	3 (2)	0.004
Postoperative infection			
Yes	6 (2.83%)	3 (3.19%)	0.86
No	206 (97.17%)	91 (96.8%)	
Hemoglobin A1c (%)			
Median (IQR)	7.9 (2.55)	6.4 (1.225)	< 0.001
Average preop RBG, mmol/L median (IQR)	10.52 (5.0834)	7.71 (2.493,225)	< 0.001
Average preop FBG, mmol/L median (IQR)	9.53 (6.325)	6.8 (3.6)	< 0.001
Number of preop days with average readings > 10 mmol/L, median (IQR)	1 (2)	0 (0.25)	< 0.001
Average postop RBG, mmol/L median (IQR)	11.3 (3.7152)	7.25 (1.87)	< 0.001
Average postop FBG, mmol/L median (IQR)	10.7 (5.2)	7.1 (2.6)	< 0.001
Inpatient treatment			
Insulin, *n* (%)	133 (62.74%)	46 (48.94%)	0.024
Metformin, *n* (%)	50 (23.58%)	27 (28.72%)	0.34
Sulfonylurea, *n* (%)	18 (8.49%)	3 (3.19%)	0.09
GLP1 agonists, *n* (%)	0 (0.00%)	0 (0.00%)	
DDP4 inhibitors, *n* (%)	22 (10.38%)	4 (4.26%)	0.08
SGLT2 inhibitors, *n* (%)	3 (1.42%)	1 (1.06%)	0.80

*Note:* DDP4 inhibitors = dipeptidyl peptidase 4 inhibitors, GLP1 agonists = glucagon-like peptide-1 agonists, postop = postoperative, preop = preoperative, SGLT2 inhibitors = sodium-glucose co-transporter-2 inhibitors.

Abbreviations: FBG = fasting blood glucose, IQR = interquartile range, POHG = postoperative hyperglycemia, RBG = random blood glucose.

**Table 3:** Infection, type of diabetes, and level of control according to postoperative infection status.

	Postoperative infection (*n* = 9)	No postoperative infection (*n* = 297)	*p* value
Type of diabetes			
Type 1, *n* (%)	1 (11.11%)	32 (10.77%)	0.97
Type 2, *n* (%)	8 (88.89%)	265 (89.23%)	
Level of control			
Controlled, *n* (%)	3 (33.33%)	132 (44.44%)	0.51
Not controlled, *n* (%)	6 (66.67%)	165 (55.56%)	

**Table 4:** Multiple logistic regression: independent variables associated with POHG.

Related variables	Hyperglycemia OR (95% CI)	*p* value
Age (years)	1.007^∗^ (0.98–1.02)	0.49
Sex, male (reference group: female)	1.66^∗^ (0.89–3.08)	0.10
BMI (kg/m^2^)	0.09 (0.01–1.04)	0.055
Average preoperative RBG during admission	1.28 (1.11–1.50)	0.001
Number of preoperative days with average readings above 10 mmol/L	1.34 (0.87–2.07)	0.18
Controlled, no (reference group: yes)	1.99 (1.05–3.75)	0.035

Abbreviations: BMI = body mass index, CI = confidence interval, OR = odds ratio, RBG = random blood glucose.

^∗^Per unit change in regressor.

## Data Availability

The data that support the findings of this study are openly available in KSAU at https://ksauhsedu-my.sharepoint.com/:x:/g/personal/421220047_ksau-hs_edu_sa/Ef-JpJwiq8tBuTnQoiYZQT8BFc5ue2jlhoN1w9uiasmApA?e=IWjnYg.
